# Treatment of acute exacerbation of idiopathic pulmonary fibrosis with direct hemoperfusion using a polymyxin B-immobilized fiber column improves survival

**DOI:** 10.1186/s12890-015-0004-4

**Published:** 2015-02-22

**Authors:** Noriyuki Enomoto, Masashi Mikamo, Yoshiyuki Oyama, Masato Kono, Dai Hashimoto, Tomoyuki Fujisawa, Naoki Inui, Yutaro Nakamura, Hideo Yasuda, Akihiko Kato, Soichiro Mimuro, Matsuyuki Doi, Shigehito Sato, Takafumi Suda

**Affiliations:** Second Division, Department of Internal Medicine, Hamamatsu University School of Medicine, 1-20-1 Handayama, Hamamatsu, 431-3192 Japan; Department of Clinical Pharmacology and Therapeutics, Hamamatsu University School of Medicine, Hamamatsu, Japan; Internal Medicine 1, Hamamatsu University School of Medicine, Hamamatsu, Japan; Blood Purification Unit, Hamamatsu University Hospital, Hamamatsu, Japan; Intensive Care Unit, Hamamatsu University Hospital, Hamamatsu, Japan; Department of Anesthesia and Resuscitation, University Hospital, Hamamatsu University School of Medicine, Hamamatsu, Japan

**Keywords:** Acute exacerbation, Idiopathic pulmonary fibrosis, Hemoperfusion, Polymyxin, PMX-DHP

## Abstract

**Background:**

Acute exacerbation of idiopathic pulmonary fibrosis (AE-IPF) has an extremely poor prognosis and there is currently no effective treatment for this condition. Direct hemoperfusion with a polymyxin B-immobilized fiber column (PMX-DHP) improves oxygenation, but it is unclear whether treatment of AE-IPF with PMX-DHP affects survival. This study elucidated the effectiveness and safety of PMX-DHP for the treatment of AE-IPF.

**Methods:**

This study included 31 patients with 41 episodes of AE-IPF. All patients received steroids. Of 31, 14 patients (20 episodes) were treated with PMX-DHP. The laboratory and physiological test results after the start of therapy and survival were retrospectively compared between patients treated with and without PMX-DHP.

**Results:**

Patients treated with PMX-DHP had a significantly greater change in PaO_2_/FiO_2_ ratio (mean ± SEM, 58.2 ± 22.5 vs. 0.7 ± 13.3, p = 0.034) and a smaller change in white blood cell count (−630 ± 959 /μL vs. 4500 ± 1190 /μL, p = 0.002) after 2 days of treatment than patients treated without PMX-DHP. The 12-month survival rate was significantly higher in patients treated with PMX-DHP (48.2% vs. 5.9%, p = 0.041). PMX-DHP was effective in patients with more severe underlying disease (GAP stages II or III; 12-month survival rate 57.1% with PMX-DHP vs. 0% without PMX-DHP, p = 0.021). Treatment with PMX-DHP was an independent predictor of better prognosis (hazard ratio 0.345, p = 0.037). Mild pulmonary thromboembolism occurred in one patient treated with PMX-DHP.

**Conclusions:**

Treatment of AE-IPF with PMX-DHP is tolerable and improves 12-month survival.

**Electronic supplementary material:**

The online version of this article (doi:10.1186/s12890-015-0004-4) contains supplementary material, which is available to authorized users.

## Background

Idiopathic pulmonary fibrosis (IPF) has a devastating prognosis [1]. Kondoh et al. first described acute exacerbation in IPF (AE-IPF) [2], which results in rapid deterioration with a significant impact on the clinical course [3-5]. The most common pathological finding in AE-IPF is diffuse alveolar damage (DAD) superimposed on usual interstitial pneumonia (UIP)-pattern [3]. There is currently no effective therapy for AE-IPF, and the prognosis is extremely poor [3,6-8]. Although steroids and immunosuppresants are used for the treatment of AE-IPF, they do not alter the natural course of the disease [8]. Novel methods of treating AE-IPF are therefore being investigated.

Direct hemoperfusion with a polymyxin B-immobilized fiber column (PMX-DHP) removes plasma endotoxins from gram-negative bacteria, and is an effective treatment for sepsis [9-11]. PMX-DHP therapy was also reported to be beneficial in patients with gram-positive bacterial infections [10,11] and endotoxin-negative infections [12]. It is noteworthy that PMX-DHP improves pulmonary oxygenation in patients with acute respiratory distress syndrome (ARDS), which is pathologically characterized by DAD [12-14]. PMX-DHP was also reported to improve oxygenation in patients with AE-IPF [15-18]. We recently reported that PMX-DHP was beneficial in patients with acute exacerbation of several types of interstitial pneumonia including IPF [19], and that a longer duration of PMX-DHP (12 hours) was more effective than a shorter duration of PMX-DHP (≤6 hours) [20]. More recently, Abe et al. reported a larger, multicenter, and retrospective study of PMX-DHP in 160 patients with acute exacerbation of interstitial pneumonia, including 73 patients with AE-IPF [16]. In their study, survival rate at one month and three months were 70.1% and 34.5%, respectively, in patients with AE-IPF, and they concluded that treatment with PMX-DHP for AE-IPF may improve the survival compared to previous reports [16]. However, the precise survival benefit of treatment of AE-IPF with PMX-DHP compared to treatment without PMX-DHP has not been fully elucidated, and the indications for using PMX-DHP are still unknown.

This study investigated the effectiveness and safety of treatment of AE-IPF with PMX-DHP, and analyzed survival after treatment with and without PMX-DHP. This study also sought to identify factors affecting survival in patients with AE-IPF.

## Methods

### Study design and subjects

Thirty-one patients who were treated for AE-IPF at our hospital between 1997 and 2013 were retrospectively reviewed. Eight patients had two episodes of AE-IPF and one patient had three episodes of AE-IPF (total, 41 episodes). Eighteen patients underwent surgical lung biopsy (SLB) before developing AE-IPF, and met the 2011 consensus criteria for IPF of the American Thoracic Society (ATS), European Respiratory Society (ERS), Japanese Respiratory Society (JRS), and Latin American Thoracic Association (ALAT) [1]. The remaining 13 patients had typical clinical and high-resolution computed tomography (HRCT) features of IPF and were diagnosed with IPF without surgical lung biopsy [21]. Patients who met the criteria for any connective tissue disorders were excluded from the study, even if their histopathological examination showed UIP. Histological diagnosis of UIP was in accordance with previously published reports [1,22].

AE-IPF was diagnosed according to the modified diagnostic criteria described by Collard et al. in 2007 [3]. Briefly, the criteria for diagnosis of AE-IPF were: 1) previous or concurrent diagnosis of IPF, 2) unexplained worsening or development of dyspnea within 30 days, 3) HRCT with new bilateral ground-glass abnormality and/or consolidation superimposed on a background reticular or honeycomb pattern, 4) no evidence of pulmonary infection, and 5) exclusion of alternative causes including left heart failure, pulmonary embolism, or an identifiable cause of acute lung injury. Serum endotoxin levels were below the level of detection in all patients treated with PMX-DHP. The study protocol was approved by the Ethical Committee of Hamamatsu University School of Medicine (approval number 25–292).

### Data collection

Clinical data, including sex, age, smoking-history, symptoms, treatments, and survival were obtained from the medical records. Laboratory and pulmonary function test results were also recorded. The severity of IPF before AE, oxygenation and HRCT findings during AE, treatments for AE, and survival were retrospectively reviewed. Disease severity of IPF within 12 months before AE was assessed using the GAP-staging system, which considers gender, age, and two lung physiology variables: forced vital capacity (FVC) and diffusion lung capacity for carbon monoxide (DLCO) [23].

### Treatments of AE-IPF

Immediately after the diagnosis of AE-IPF, all patients were started on high-dose corticosteroid pulse therapy (methylprednisolone 1,000 mg/day for 3 days) followed by a tapering dose of prednisolone with or without immunosuppresants (cyclophosphamide or cyclosporine). Some patients also received intravenous sivelestat sodium hydrate.

### PMX-DHP therapy

**Figure 1:**
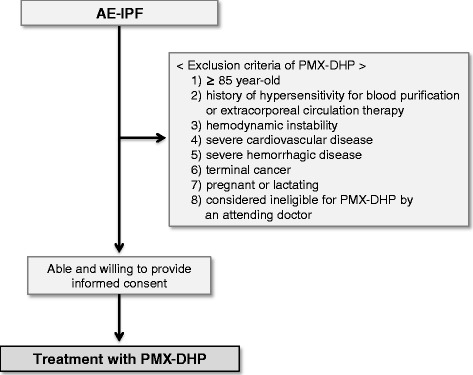
**A flow chart of treatment with PMX-DHP.** Patients who were diagnosed as AE-IPF received treatment with PMX-DHP according to inclusion and exclusion criteria as written in “Methods” section.

Some patients were treated with PMX-DHP (PMX; Toray Medical Co., Ltd, Tokyo, Japan) starting at the same time as the corticosteroid and immunosuppressant therapy. Starting in 2006, patients were treated with PMX-DHP whenever practicable. Inclusion criteria of PMX-DHP for the treatment of AE-IPF were as follows: 1) current diagnosis of AE-IPF, 2) able and willing to provide informed consent. Exclusion criteria of PMX-DHP for the treatment of AE-IPF were as follows: 1) ≥ 85 year-old, 2) history of hypersensitivity for blood purification or extracorporeal circulation therapy, 3) hemodynamic instability, 4) severe cardiovascular disease, 5) severe hemorrhagic disease, 6) terminal cancer, 7) pregnant or lactating, 8) considered ineligible for PMX-DHP by an attending doctor. A flow chart of treatment with PMX-DHP according to these inclusion and exclusion criteria is shown in Figure 1. A total of 14 patients (20 episodes) were treated with PMX-DHP. A double-lumen catheter was placed via a femoral or internal jugular vein, and PMX-DHP was administered for 6 or 12 hours (usually 12 hours) at a flow rate of 80–100 mL/minute. PMX-DHP was subsequently repeated once or twice (usually once) within 24 hours. Nafamostat mesilate and/or heparin sodium were used for anticoagulant therapy.

### Measurement of cytokine concentrations

Blood samples were collected before and after PMX-DHP. The serum level of angiopoietin-2 was measured using enzyme-linked immunosorbent assay (R&D Systems, Inc., MN, USA).

### Evaluation of HRCT findings

The extent of lung opacities was measured on three HRCT slices: at the bifurcation of the trachea, at the bases of the lower lobes, and at the midpoint between the other two slices. The extent of fibrosis in each lobe was scored using following system: 0, none; 1, 1–10%; 2, 11–25%; 3, 26–50%; 4, 51–75%; and 5, 76–100%. The sum of the scores from five lobes (0–25) was used to express the extent of reticular opacity, honeycombing, consolidation opacity, and ground-glass opacity (GGO) in each patient. The pattern of AE-IPF on HRCT was classified as 1) peripheral, 2) multifocal, or 3) diffuse as reported by Akira [24]. The HRCT findings were reviewed by two observers, and the rate of agreement between observers were evaluated by weighted-kappa coefficients. The coefficients were between 0.52 and 0.56. If the score differed between observers, a consensus was reached after discussion.

### Statistical analysis

Statistical analyses were performed using StatView J-4.5 (SAS Institute Inc; Cary, NC, USA). Categorical data were compared using the Chi-square test or Fisher’s exact probability test for independence, and continuous data were compared using the paired *t*-test. Overall survival of patient groups was estimated using Kaplan-Meier curves, and was compared between groups using the log-rank test. The relationships between variables, including treatment with PMX-DHP and mortality, were evaluated using Cox’s proportional hazards regression analysis. All tests were two-sided and statistical significance was set at p < 0.05.

## Results

### Demographic, laboratory, and physiological data

The clinical characteristics of the patients, including the laboratory and physiological test results at the time of the first AE-IPF, are shown in Table 1. The median age of the patients was 69 years, and 28 patients were male. The median observation period was 53 months. The percent predicted FVC (%FVC) and percent predicted DLCO (%DLCO) within 12 months before AE-IPF were moderately impaired (median, 58.8% and 57.1%, respectively). The minimum SpO_2_ and the distance walked in the 6-minute walk test (6MWT) were also impaired (median, 82% and 360m, respectively). The severity of IPF within 12 months before AE-IPF was staged using the GAP system [23]. The most common GAP grade was III (the most severe stage), accounting for 10 patients (32.3%). Eighteen of 31 patients (58.1%) received treatment for IPF before AE-IPF, including steroids, immunosuppresants, and pirfenidone. Nine patients (29.0%) received long-term oxygen therapy before AE-IPF. The mortality rate was 38.7% at 3 months after AE-IPF and 74.2% at 12 months after AE-IPF. Fourteen patients (45.2%) were treated with PMX-DHP for 20 episodes of AE-IPF and 17 patients (54.8%) were treated without PMX-DHP for 21 episode of AE-IPF. There were 6 patients treated without PMX-DHP even after 2006 (2009–2012). One of them was aged 84-year old and had advanced-lung cancer. Another patient had severe cardiac disease. Another three patients received long-term oxygen therapy and had poor activity of daily living due to slowly progressive IPF before AE-IPF. Therefore, these 5 patients were considered ineligible for PMX-DHP. The other one patient showed good response to treatment with steroids and did not need further PMX-DHP.

**Table 1:** **Clinical characteristics, laboratory and physiological test results in all patients with IPF**

	**Median (range), n = 31**
Age, years	69 (50, 84)
Sex, male/female	28/3
Smoking, never/ex/current	4/23/4
Pack-year of smoking	35 (0, 81)
Diagnosis, surgical lung biopsy/clinical, n	18/13
Period from symptom onset, m	49 (0, 203)
Observation period, m	53 (2, 205)
%FVC before AE, %	58.8 (37.5, 89.3)
FEV1% before AE, %	87.4 (78.5, 107.0)
%DLCO before AE, %	57.1 (33.5, 88.2)
PaO_2_ at rest before AE, Torr	70.5 (49.0, 91.0)
6MWT distance before AE, m	360 (160, 507)
6MWT minSpO_2_ before AE, %	82 (60, 87)
Serum LDH at AE, ng/mL	344 (220, 602)
Serum KL-6 at AE, U/mL	1367 (481, 6404)
Serum SP-D at AE, ng/mL	339 (23, 966)
Extent scores on HRCT before AE (full score: 25)	12 (7, 19)
The GAP staging system before AE, I/II/III/unknown	8/7/10/6
Preceding treatments for IPF, +/ -	18/13
Preceding oxygen therapy, +/ -	9/22
Period from admission to commencement of treatments for AE, day	1 (0, 17)
Treatment with PMX-DHP, +/-	14/17
Mortality 3 months after AE, n (%)	12 (38.7)
Mortality 12 months after AE, n (%)	23 (74.2)

AE = acute exacerbation; IPF = idiopathic pulmonary fibrosis; FVC = forced vital capacity;

FEV1 = forced expiratory volume in 1 second; DLCO: diffusion lung capacity for carbon monoxide; 6MWT = six-minute walk test; LDH = lactate dehydrogenase; KL-6 = Krebs von den Lungen-6; SP-D = surfactant protein D; PMX-DHP = direct hemoperfusion with a polymyxin B-immobilized fiber column.

### Comparisons between patients treated with and without PMX-DHP

The demographic data, preceding IPF treatment, laboratory and physiological tests results, HRCT findings, and treatment for AE-IPF in patients treated with and without PMX-DHP are shown in Table 2. There were no significant differences between the two groups. Although patients treated with PMX-DHP tended to have a lower GAP-stage before AE-IPF, there were no significant differences before AE-IPF in PaO_2_ at rest, 6MWT results, serum levels of Krebs von den Lungen-6 (KL-6) and surfactant protein D (SP-D), or extent score based on HRCT between the two groups. There were also no significant differences in PaO_2_/FiO_2_ (P/F) ratio at the time of AE-IPF, time from admission to the start of treatment for AE-IPF, HRCT-patterns at the time of AE-IPF, administration of corticosteroids, immunosuppresants and other agents, or intubation rate between the two groups. Serum endotoxin levels were below the level of detection in all patients treated with PMX-DHP.

**Table 2:** **Comparisons between patients treated with and without PMX-DHP**

	**PMX-DHP –(median (range), n = 17)**	**PMX-DHP + (median (range), n = 14)**	**p value**
Age, years	71 (50, 84)	66.5 (52, 81)	0.109
Sex, male/female	15/2	13/1	0.999
Smoking, never/ex/current	3/13/1	1/10/3	0.346
Pack-year of smoking	25 (0, 80)	45 (0, 81)	0.086
Period from symptom onset to AE, mo	72 (0, 203)	30 (2, 156)	0.082
Observation period, mo	66 (9, 205)	50 (2, 162)	0.463
%FVC before AE, %	46.7 (37.5, 89.3)	61.9 (40.7, 88.0)	0.136
FEV1% before AE, %	87.5 (79.7, 100.0)	87.4 (78.5, 107.0)	0.769
%DLCO before AE, %	57.1 (33.5, 88.2)	62.0 ( 44.0, 77.8)	0.892
PaO_2_ at rest before AE, Torr	68.5 (53.9, 86.6)	72.8 (49.0, 91.0)	0.978
6MWT distance before AE, m	375 (160, 465)	365 (175, 507)	0.523
6MWT minSpO_2_ before AE, %	82 (62, 83)	82 (60, 87)	0.721
Serum LDH at AE, IU/L	322 (220, 601)	347 (277, 602)	0.409
Serum KL-6 at AE, U/mL	1367 (634, 3160)	1690 (481, 6404)	0.174
Serum SP-D at AE, ng/mL	295 (24, 645)	469 (102, 966)	0.159
Extent scores on HRCT before AE (full score: 25)	11.5 (7, 19)	13 (7, 15)	0.624
HRCT-patterns at AE, peripheral/multifocal/diffuse/unknown	1/2/13/1	3/1/10/0	0.483
The GAP staging system before AE, I/II/III/unknown	2/3/7/5	6/5/3/0	0.059
Preceding treatment for IPF, +/-	10/7	8/6	0.925
Preceding oxygen therapy, +/-	5/12	4/10	0.999
P/F ratio at AE	162 (44, 316)	171 (31, 368)	0.741
Extent scores on HRCT at AE (full score: 25)	20 (13, 25)	20.5 (14, 25)	0.915
Period from admission to commencement of treatments for AE, day	1 (0, 6)	0.5 (0, 17)	0.755
Administration of steroid-pulse therapy for AE, +/-	17/0	14/0	NS.
Administration of Immunosuppresants for AE, +/-	12/5	9/5	0.709
Administration of Sivelestat sodium hydrate for AE, +/-	3/14	5/9	0.413
Intubation at AE, +/-	3/14	5/9	0.413

PMX-DHP = direct hemoperfusion with a polymyxin B-immobilized fiber column; AE = acute exacerbation; IPF = idiopathic pulmonary fibrosis; FVC = forced vital capacity; FEV1 = forced expiratory volume in 1 second; DLCO = diffusion lung capacity for carbon monoxide; 6MWT = six-minute walk test; LDH = lactate dehydrogenase; KL-6 = Krebs von den Lungen-6; SP-D = surfactant protein D; HRCT = high-resolution computed tomography; P/F = PaO_2_/FiO_2._

### PMX-DHP

The details of PMX-DHP are shown in Table 3. Eighteen of 20 episodes of AE-IPF (90%) were treated with 12-hour periods of PMX-DHP and the remaining 2 episodes (10%) were treated with 6-hour periods of PMX-DHP. Nineteen episodes (95%) of AE-IPF were treated with two periods of PMX-DHP and the other episode was treated with three periods of PMX-DHP. The median time from admission to the start of PMX-DHP was one day. The anticoagulants administered were nafamostat mesilate for 17 episodes of AE-IPF and heparin sodium for 3 episodes of AE-IPF. Thrombomodulin-α was administered to one patient, who did not receive PMX-DHP. Mild pulmonary thromboembolism occurred in one patient, which resolved after anticoagulant therapy. No other adverse events were observed, and PMX-DHP therapy was tolerable in all patients.

**Table 3:** **Details of PMX-DHP and adverse events**

	**PMX-DHP (n = 14, 20 episodes)**
Perfusion duration, 6h/12h	2/18
Cycles of perfusion, 2 times/3 times	19/1
Interval of each perfusion, 12h/18h	18/2
Period from admission to commencement of PMX-DHP, day	1 (0, 17)
Adverse events	Pulmonary thromboembolism: 1

PMX-DHP = direct hemoperfusion with a polymyxin B-immobilized fiber column.

### Effects of treatment with PMX-DHP on acute phase of AE-IPF

**Figure 2:**
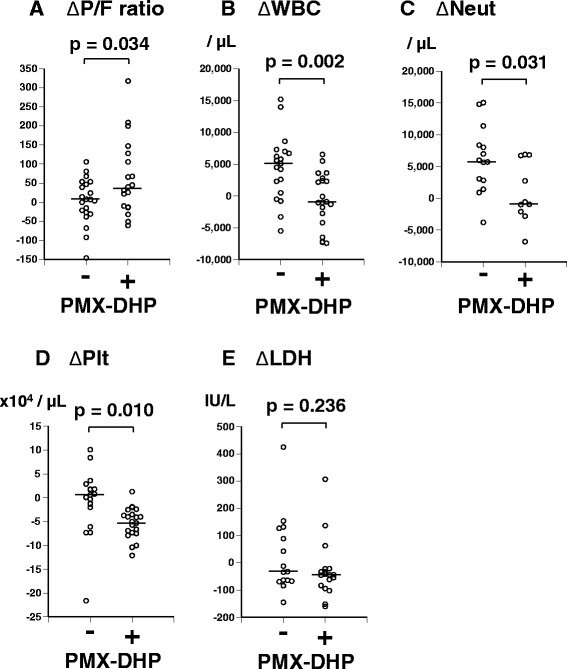
**Comparisons after 2 days of treatment between patients treated with and without PMX-DHP. A)** Changes in PaO_2_/FiO_2_ ratio (ΔP/F ratio; P/F ratio 2 days after the start of treatments – P/F ratio just before treatments). **B)** Changes in the white blood cell count (ΔWBC). **C)** Changes in the neutrophil count (ΔNeut). **D)** Changes in the platelet count (ΔPlt). **E)** Changes in serum lactate dehydrogenase level (ΔLDH). There were significant differences in ΔP/F ratio, ΔWBC, ΔNeut, and ΔPlt (all p < 0.05). The horizontal bars indicate the median values.

**Figure 3:**
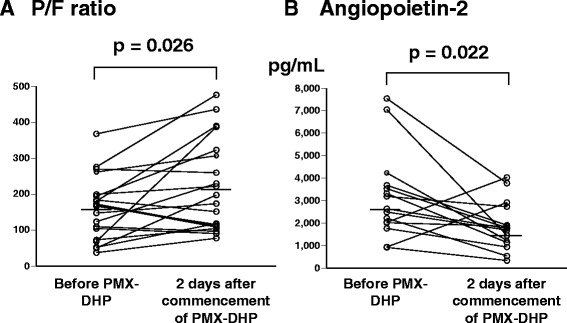
**Changes in PaO**_**2**_ **/FiO**_**2**_ **ratio and angiopoietin-2 after 2 days of treatment in patients treated with PMX-DHP. A)** Changes in PaO_2_/FiO_2_ (P/F) ratio. **B)** Changes in the serum level of angiopietin-2 level. Both parameters were significantly different after treatment (both p < 0.05). The horizontal bars indicate the median values.

Changes after 2 days of treatment were compared between patients treated with and without PMX-DHP (Figure 2). In the PMX-DHP group, steroid-pulse therapy and PMX-DHP were started simultaneously in 17 of the 20 episodes (85.0%). Change in P/F ratio (ΔP/F ratio; P/F ratio 2 days after the start of treatments – P/F ratio just before treatments) was significantly greater in patients treated with PMX-DHP than in patients treated without PMX-DHP (mean ± standard error of the mean, 58.2 ± 22.5 vs. 0.7 ± 13.3, p = 0.034; Figure 2A). Patients treated with PMX-DHP also had a significantly smaller change in white blood cell count (−630 ± 959/μL vs. 4500 ± 1190 /μL, p = 0.002; Figure 2B) and change in neutrophil count (1095 ± 1649/μL vs. 6166 ± 1406/μL, p = 0.031; Figure 2C) than patients treated without PMX-DHP. Patients treated with PMX-DHP also had a significant smaller change in platelet count than patients treated without PMX-DHP (−5.4 ± 0.7 × 10^4^/μL vs. -0.9 ± 1.5 × 10^4^/μL, p = 0.010; Figure 2D), and there were no episodes of bleeding. There was no significant difference in the change in serum lactate dehydrogenase level between the two groups (Figure 2E). In patients treated with PMX-DHP, P/F ratio increased significantly after 2 days (p = 0.026; Figure 3A) and the serum angiopietin-2 level, which is related to vascular permeability [25], decreased significantly after 2 days (*p* = 0.022; Figure 3B).

### Effects of treatment with PMX-DHP on survival

**Figure 4:**
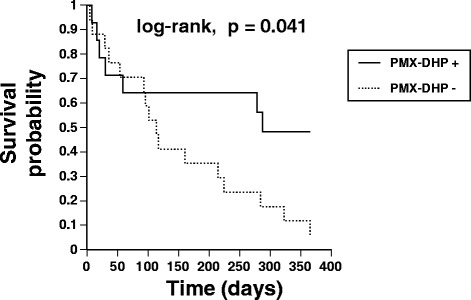
**Kaplan-Meier survival curves showing the effects of treatment of AE-IPF with PMX-DHP on survival.** Patients treated with PMX-DHP had significantly better survival than patients treated without PMX-DHP (48.2% vs. 5.9%; log-rank test, p = 0.041).

Twenty -three of the 31 patients (74.2%) died within 12 months of the first episode of AE-IPF. Eighteen patients died of respiratory failure, four died of infection, and one died of an unknown cause. The Kaplan-Meier survival curves from the time of AE-IPF are shown in Figure 4. The 12-month survival rate was significantly better in patients treated with PMX-DHP than in patients treated without PMX-DHP (48.2% vs. 5.9%, log-rank test, p = 0.041). Interestingly, the survival benefit of treatment with PMX-DHP was evident from 3 months after the time of AE-IPF. The results of univariate Cox proportional hazards analyses to identify factors that predicted prognosis are shown in Table 4. Treatment with PMX-DHP was significantly associated with a more favorable prognosis (hazard ratio [HR] 0.399, p = 0.047). The prognosis was also significantly associated withΔP/F ratio (HR 0.990, p = 0.008) andΔLDH (HR 1.006, p = 0.009). Multivariate Cox proportional hazards analysis adjusted for GAP stage (which considers age, %FVC, and %DLCO) showed that the prognosis was independently associated with treatment with PMX-DHP (HR 0.345, p = 0.037), ΔP/F ratio (HR 0.989, p = 0.004), andΔLDH (HR 1.007, p = 0.007) (Table 5).

**Table 4:** **Univariate Cox proportional hazards analyses for survival**

**Variable**	**Hazard ratio**	**95% CI**		**p value**
		**Lower**	**Upper**	
Age at biopsy, yr	1.040	0.977	1.108	0.220
Sex, female	0.432	0.122	1.526	0.193
Pack-year of smoking	0.993	0.978	1.008	0.367
Symptom onset, mo	1.001	0.994	1.009	0.737
Extent score on HRCT before AE	1.127	0.970	1.311	0.119
FVC before AE, % pred	0.989	0.956	1.023	0.514
FEV_1_/FVC, %	0.953	0.877	1.035	0.249
DLCO before AE, % pred	0.987	0.958	1.018	0.416
Resting PaO_2_, mmHg	0.999	0.961	1.039	0.961
Distance in six-minute walk test	0.998	0.992	1.005	0.609
Minimum SpO_2_ in six-minute walk test	0.960	0.884	1.043	0.338
Serum LDH, IU/L	1.003	0.997	1.008	0.305
Serum KL-6, U/mL	1.000	1.000	1.000	0.447
Serum SP-D, ng/mL	0.999	0.997	1.001	0.256
The GAP staging system before AE	1.089	0.639	1.854	0.754
Preceding treatments for IPF, +	0.913	0.399	2.088	0.829
Preceding oxygen therapy, +	1.662	0.678	4.074	0.267
P/F ratio at AE	0.999	0.994	1.003	0.565
Extent score on HRCT at AE	1.077	0.954	1.215	0.231
Period from admission to commencement of treatments for AE, day	1.104	0.985	1.237	0.090
Administration of Immunosuppressant for AE, +	1.403	0.548	3.593	0.481
Administration of Sivelestat sodium hydrate for AE, +	0.787	0.290	2.131	0.637
PMX-DHP, +	0.399	0.161	0.988	0.047
ΔWBC 2 days after commencement of treatments for AE, /μL	1.000	1.000	1.000	0.423
ΔNeutrophil 2 days after commencement of treatments for AE, /μL	1.000	1.000	1.000	0.607
ΔP/F ratio 2 days after commencement of treatments for AE	0.990	0.983	0.997	0.008
ΔLDH 2 days after commencement of treatments for AE, IU/L	1.006	1.002	1.011	0.009
ΔAngiopoietin-2 2 days after commencement of treatments for AE, pg/mL	1.000	1.000	1.001	0.307

AE = acute exacerbation; IPF = idiopathic pulmonary fibrosis; FVC = forced vital capacity;

FEV1 = forced expiratory volume in 1 second; DLCO = diffusion lung capacity for carbon monoxide; 6MWT = six-minute walk test; LDH = lactate dehydrogenase; KL-6 = Krebs von den Lungen-6; SP-D = surfactant protein D; P/F = PaO_2_/FiO_2_; HRCT = high-resolution computed tomography; PMX-DHP = direct hemoperfusion with a polymyxin B-immobilized fiber column.

**Table 5:** **Multivariate Cox proportional hazards analysis for survival adjusted for GAP-stage**

**Variable**	**Hazard ratio**	**95% CI**		**p value**
		**Lower**	**Upper**	
Extent score on HRCT before AE	1.177	0.982	1.409	0.078
Period from admission to commencement of treatments for AE, day	1.092	0.974	1.224	0.132
PMX-DHP, +	0.345	0.127	0.936	0.037
ΔP/F ratio 2 days after commencement of treatments for AE	0.989	0.982	0.997	0.004
ΔLDH 2 days after commencement of treatments for AE, IU/L	1.007	1.002	1.013	0.007

AE = acute exacerbation; HRCT = high-resolution computed tomography; PMX-DHP = direct hemoperfusion with a polymyxin B-immobilized fiber column; P/F = PaO_2_/FiO_2_; LDH = lactate dehydrogenase.

**Figure 5:**
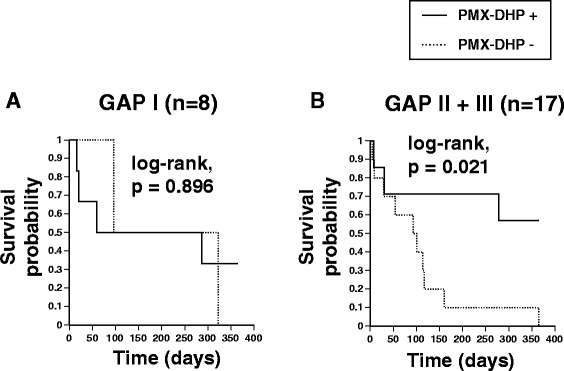
**Survival curves according to the severity of IPF during the 12 months before AE-IPF. A)** Patients with GAP stage I disease. **B)** Patients with GAP-stage II or III disease (log-rank test, p = 0.021). In patients with more severe disease (GAP-stage II or III), the 12-month survival rate was significantly higher in those who were treated with PMX-DHP than in those who were treated without PMX-DHP (57.1% vs. 0%).

Survival curves according to the severity of disease before AE-IPF showed that patients with less severe IPF (GAP-stage I) did not have a significant benefit from treatment with PMX-DHP (PMX-DHP+: HR 0.892, p = 0.896; log-rank test, p = 0.896; Figure 5A), whereas patients with more severe IPF (GAP-stage II and III) had significantly better survival if they were treated with PMX-DHP (PMX-DHP+: HR 0.226, p = 0.031; log-rank test, p = 0.021; Figure 5B). In patients with GAP-stage II or III disease, the 12-months survival rates was 57.1% in patients treated with PMX-DHP and 0% in patients treated without PMX-DHP. In this study, 6 patients without PMX-DHP treatment satisfied exclusion criteria. To reduce this bias in non-PMX group, we excluded these 6 patients applying exclusion criteria from the analysis. We compared survival curves between 14 patients with PMX-DHP treatment and 11 patients without PMX-DHP treatment, who did not meet exclusion criteria. Although the difference was not significant, patients treated with PMX-DHP tended to have better survival than those treated without PMX-DHP (Additional file 1: Figure S1, 12-month survival rate, 48.2% vs. 9.1%; log-rank test, p = 0.115). Furthermore, in 12 patients with GAP-stage II or III disease who did not meet exclusion criteria, patients had a significant benefit from treatment with PMX-DHP (Additional file 2: Figure S2, 12-month survival rate, 57.1% vs. 0%; log-rank test, p = 0.048).

## Discussion

Acute exacerbation is common in patients with IPF, with a reported rate of 8.6-14.2% per year [4,8]. Acute exacerbation has a significant impact on survival, and contributes to the grim prognosis of IPF [1,4,8]. There is currently no effective treatment for AE-IPF, including steroid therapy [8], and the prognosis of AE-IPF is extremely poor [3,5-8]. Development of a novel method of treatment for IPF-AE is therefore desirable. This study found that treatment for AE-IPF with PMX-DHP was safe without severe adverse events, and that 12-month survival rate was significantly higher in patients treated with PMX-DHP than in patients treated without PMX-DHP. The effects of treatment with PMX-DHP were evident in patients with a more severe stage of IPF, and treatment of AE-IPF with PMX-DHP was associated with a favorable outcome.

We previously reported the beneficial effects of treatment with PMX-DHP for acute exacerbation of several interstitial pneumonias including AE-IPF, and improved oxygenation in patients treated with PMX-DHP [19]. More recently, Abe et al. reported a larger, multicenter, and retrospective study, which found that treatment with PMX-DHP may improve the survival in patients with AE-IPF compared to previous reports [16]. The precise mechanism by which PMX-DHP improves oxygenation in patients with AE-IPF is unknown. Abe et al. reported that activated neutrophils were adsorbed by the PMX-column [26], and that the white blood cells count decreased after PMX-DHP [16]. The present study also found significantly decreased white blood cell and neutrophil counts in patients treated with PMX-DHP than in patients treated without PMX-DHP. Treatment of AE-IPF or septic shock with PMX-DHP has also been reported to decrease the concentrations of serum matrix metalloproteinase (MMP)-9 [26], vascular endothelial growth factor (VEGF) [27], and angiopoietin-2 [28,29], which can facilitate vascular permeability. Direct interactions between activated neutrophils and endothelial cells can induce production of angiopoietin-2 by endothelial cells [30]. In the present study, the serum level of angiopoietin-2 was significantly decreased after treatment with PMX-DHP. These treatment effects may be associated with changes in vascular permeability. The relationships between PMX-DHP and factors that facilitate vascular permeability need to be further investigated.

To date, the effect of treatment of AE-IPF with PMX-DHP on their survival has been unclear. The previously reported 12-month survival rate after AE-IPF was about 20% [7,8]. In the present study, the 12-month survival rate was 48.2%, and survival was significantly better in patients treated with PMX-DHP than in patients treated without PMX-DHP. To the best of our knowledge, this is the first study to show that treatment of AE-IPF with PMX-DHP significantly improves survival compared to that without PMX-DHP.

Abe et al. reported a 3-month survival rate of 34.5% in patients treated for AE-IPF with PMX-DHP, which is better than in previous reports [16]. The present study found an even higher 3-month survival rate (61.3%) after treatment with PMX-DHP. This may be because of differences in the duration and timing of PMX-DHP between the two studies. Most patients in the present study received 12-hour periods of PMX-DHP, whereas most patients in the study by Abe et al. received 6-hour periods of PMX-DHP. We recently reported that a longer duration of PMX-DHP (12 hours) was more effective than a shorter duration of PMX-DHP (≤6 hours) [20]. As for timing, time from hospital admission to the start of PMX-DHP was short in the present study (median, one day). PMX-DHP was started as soon as possible in this study, at the same time as steroid-pulse therapy in 17 of the 20 episodes (85.0%), whereas PMX-DHP was started after the start of steroid therapy in the majority of patients (98%) in the study by Abe et al. [16]. These differences in the duration and timing of PMX-DHP may have resulted in better survival in the present study.

Interestingly, this study showed a difference in survival from 3 months after the start of treatment for AE-IPF. The mechanisms underlying this phenomenon are unknown. Oishi et al. reported that PMX-fiber removed profibrotic cytokines such as fibroblast growth factor (FGF), platelet-derived growth factor (PDGF), and transforming growth factor-β (TGF-β) in patients with AE-IPF [27]. Therefore, removal of these profibrotic cytokines may have improved the survival rate in fibrotic phase in patients treated with PMX-DHP. Alternatively, only survived patients, whose pulmonary reserve capacity strikingly decreased, at 3 months from the onset of AE-IPF may have been benefited from treatment with PMX-DHP. Further studies are needed to clarify the mechanisms underlying these findings.

The severity of IPF before AE-IPF was evaluated using the GAP staging system. The GAP system is an easy and reliable method of staging the severity of IPF [23], that considers of gender, age, and two lung physiology variables (%FVC and %DLCO). In the present study, patients with more severe disease (GAP stage II or III) had better survival after treatment with PMX-DHP. This result may imply an early treatment of AE-IPF with PMX-DHP in patients with advanced IPF. However, patients with less severe disease (GAP stage I) consist of only eight, and further study is needed to clarify this issue.

In the analysis of prognostic factors, PMX-DHP, ΔP/F ratio, and ΔLDH were significantly associated with a more favorable prognosis. However, there was no difference in ΔLDH between PMX and non-PMX groups. We suspect that slight hemolysis may have occurred in patients treated with PMX-DHP, and this hemolysis may be why ΔLDH was equivalent between PMX and non-PMX groups.

This study has a number of limitations. First, only a small number of patients with AE-IPF were included. Second, the data were collected retrospectively. Third, the treatment for AE-IPF other than PMX-DHP and steroids was not uniform. Finally, AE-IPF in a Japanese population may not be the same as that in other ethnic groups [31]. AE-IPF is the most common cause of death in Japanese patients with IPF (40%) [32], and this incidence is higher than that in western countries (30%) [33]. This discrepancy in the incidence of AE-IPF may indicate an ethnic difference. Therefore, the response to treatment with PMX-DHP may be different between Japanese and other ethnic groups. A larger and prospective cohort study should therefore be conducted to further evaluate the treatment of AE-IPF with PMX-DHP.

## Conclusions

The present study found that treatment of AE-IPF with PMX-DHP was safe, was not associated with severe adverse events, and improved oxygenation. The 12-month survival rate was better in patients treated with PMX-DHP than in patients treated without PMX-DHP. The effects of treatment with PMX-DHP were evident in patients with a more severe stage of IPF. Treatment with PMX-DHP was also a favorable prognostic factor in patients with AE-IPF. Recent randomized controlled trials reported promising results using novel medications for chronic stage of IPF, such as nintedanib and pirfenidone [34,35]. However, there is still no effective treatment for AE-IPF, which worsens the overall prognosis of IPF [4]. Treatment of AE-IPF with PMX-DHP may improve the clinical course of this severe condition, and may improve the overall survival of patients with IPF. A larger prospective controlled trial is needed to further evaluate the treatment of AE-IPF with PMX-DHP.

## Additional files

Additional file 1: Figure S1. Kaplan-Meier survival curves in patients who did not satisfy exclusion criteria of PMX-DHP. Six patients who satisfied exclusion criteria of PMX-DHP were excluded from the survival analysis. In 25 patients with AE-IPF, although the difference was not significant, patients treated with PMX-DHP tended to have better survival (12-month survival rate, 48.2% vs. 9.1%; log-rank test, p = 0.115). Additional file 2: Figure S2. Survival analysis in patients with GAP-stage II or III disease who did not satisfy exclusion criteria of PMX-DHP. In 12 patients with GAP-stage II or III disease who did not meet exclusion criteria, patients had a significant benefit from treatment with PMX-DHP (12-month survival rate, 57.1% vs. 0%; log-rank test, p = 0.048).
