# Supplementary figures and images for: Treatment of acute exacerbation of idiopathic pulmonary fibrosis with direct hemoperfusion using a polymyxin B-immobilized fiber column improves survival
Source: BMC Pulm Med. 2015 Feb 22;15:15. doi: 10.1186/s12890-015-0004-4 (PMC4349669; doi:10.1186/s12890-015-0004-4)

## Slide 1
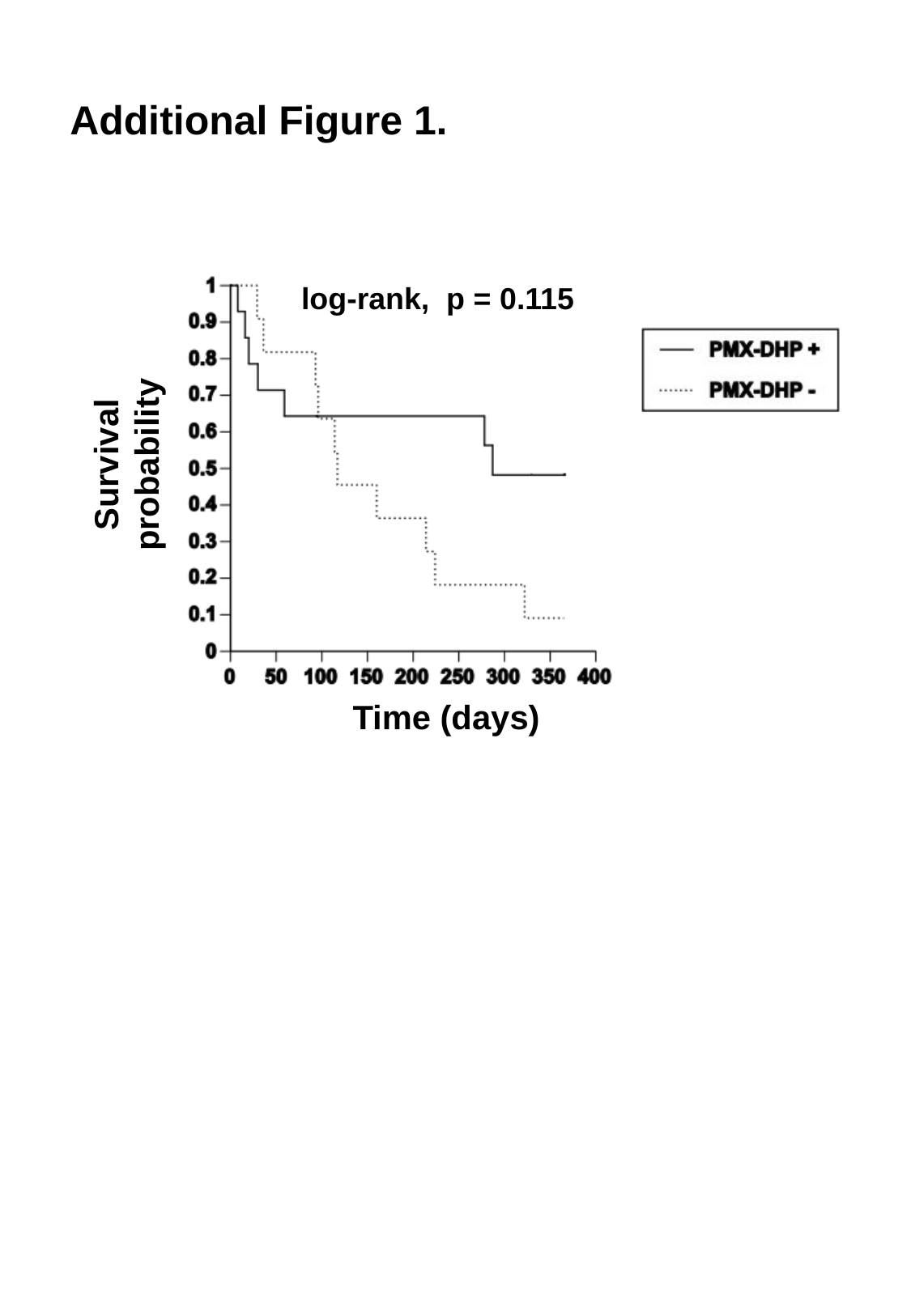

Additional Figure 1.
log-rank, p = 0.115
Survival probability
Time (days)

Supplement: Additional file 1: Figure S1. — Kaplan-Meier survival curves in patients who did not satisfy exclusion criteria of PMX-DHP. Six patients who satisfied exclusion criteria of PMX-DHP were excluded from the survival analysis. In 25 patients with AE-IPF, although the difference was not significant, patients treated with PMX-DHP tended to have better survival (12-month survival rate, 48.2% vs. 9.1%; log-rank test, p = 0.115). [file 12890_2015_4_MOESM1_ESM.pptx]

## Slide 1
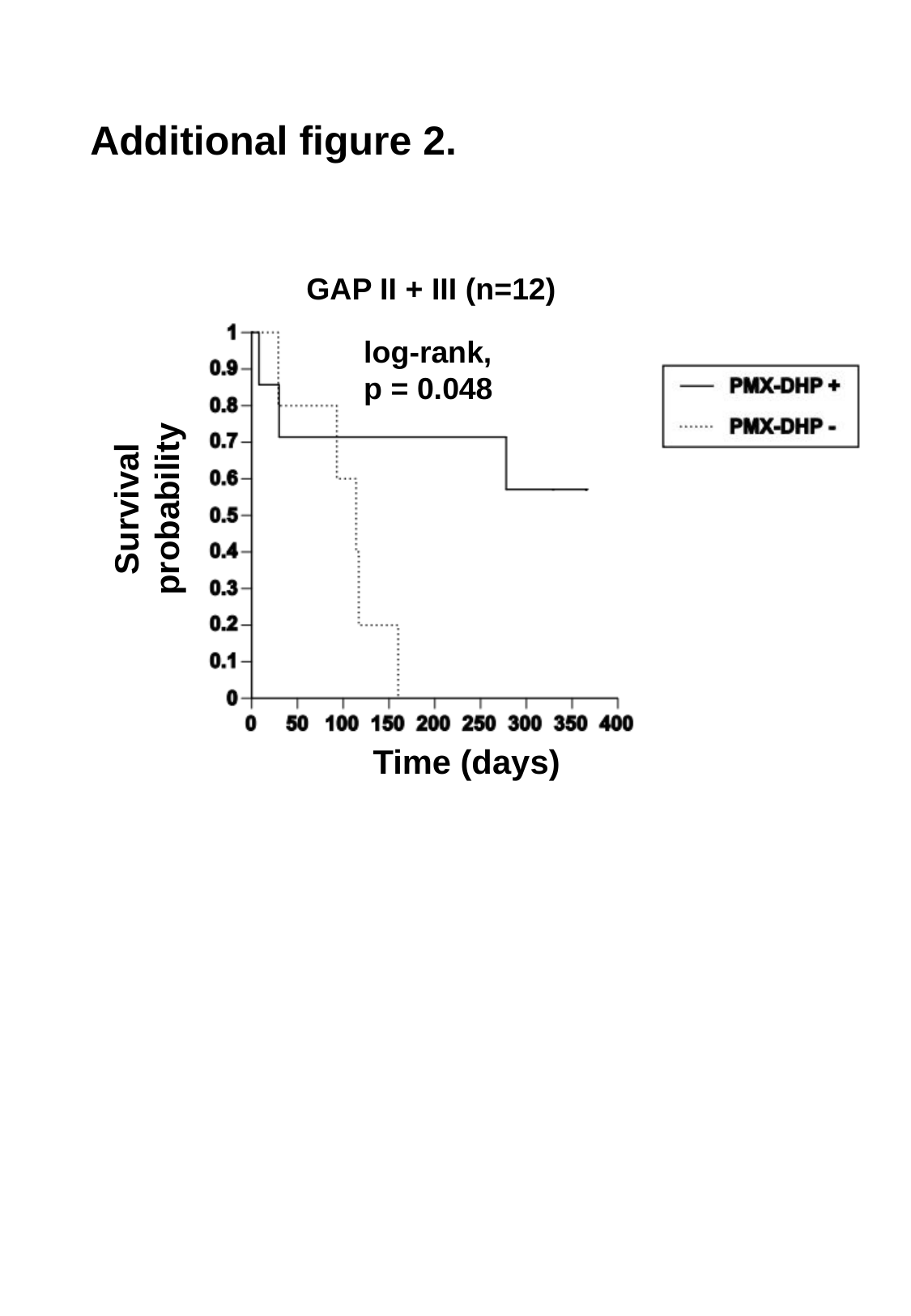

Additional figure 2.
GAP II + III (n=12)
log-rank,
p = 0.048
Survival probability
Time (days)

Supplement: Additional file 2: Figure S2. — Survival analysis in patients with GAP-stage II or III disease who did not satisfy exclusion criteria of PMX-DHP. In 12 patients with GAP-stage II or III disease who did not meet exclusion criteria, patients had a significant benefit from treatment with PMX-DHP (12-month survival rate, 57.1% vs. 0%; log-rank test, p = 0.048). [file 12890_2015_4_MOESM2_ESM.pptx]
